# Diversity of Cardiologic Issues in a Contemporary Cohort of Women With Breast Cancer

**DOI:** 10.3389/fcvm.2021.654728

**Published:** 2021-10-01

**Authors:** Giacomo Tini, Pietro Ameri, Giulia Buzzatti, Matteo Sarocchi, Roberto Murialdo, Giulia Guglielmi, Eleonora Arboscello, Alberto Ballestrero, Lucia Del Mastro, Paolo Spallarossa, Italo Porto

**Affiliations:** ^1^Cardiovascular Disease Unit, Istituto di Ricovero e Cura a Carattere Scientifico (IRCCS) Ospedale Policlinico San Martino, Genova, Italy; ^2^Department of Internal Medicine, University of Genova, Genova, Italy; ^3^Breast Unit, Istituto di Ricovero e Cura a Carattere Scientifico (IRCCS) Ospedale Policlinico San Martino, Genova, Italy; ^4^Internal Medicine Unit, Istituto di Ricovero e Cura a Carattere Scientifico (IRCCS) Ospedale Policlinico San Martino, Genova, Italy; ^5^Department of Emergency, Istituto di Ricovero e Cura a Carattere Scientifico (IRCCS) Ospedale Policlinico San Martino, Genova, Italy

**Keywords:** women, cardiovascular health, Cardio-Oncology, breast cancer, cardiotoxicity

## Abstract

**Background:** Women with breast cancer (BC) represent a special population particularly exposed to cardiovascular disease (CVD) risk. However, cardiologic assessment in BC is mostly limited to detection of left ventricular dysfunction cardiotoxicity (LVD-CTX) due to anticancer treatments. Our aim was to comprehensively investigate CV profile and events in a contemporary BC cohort.

**Methods and Results:** Records of BC patients referred for a Cardio-Oncologic evaluation before starting anticancer treatments, between 2016 and 2019, were retrospectively reviewed (*n* = 508). Information regarding prevalence and control of CV risk factors, and novel CVD diagnoses were extracted. Occurrence of LVD-CTX, CV events other than LVD-CTX and mortality was assessed. Mean age of study population was 64 ± 13 years; 287 patients were scheduled to receive anthracycline and 165 anti-HER2 therapy. Overall, 53% of BC women had ≥2 CV risk factors, and 67% had at least one of arterial hypertension, dyslipidaemia or diabetes mellitus not adequately controlled. Eighteen (4%) patients were diagnosed a previously unknown CVD. Over a mean follow-up of 2.5 ± 1 years, 3% of BC patients developed LVD-CTX, 2% suffered from other CV events and 11% died. CV risk factors were not associated with LVD-CTX, except for family history of CAD. On the contrary, patients with other CV events exhibited a worse CV profile. Those who died more commonly experienced CV events other than LVD-CTX (*p* = 0.02).

**Conclusions:** BC women show a suboptimal CV risk profile and are at risk of CV events not limited to LVD-CTX. A baseline Cardio-Oncologic evaluation was instrumental to implement CV prevention and to optimize CV therapies.

## Background

Awareness toward cardiovascular disease (CVD) remains low among women, even if it is the leading cause of mortality and morbidity in the female sex (1, 2). Patients with breast cancer (BC) represent a special female population particularly exposed to CV risk (2). Indeed, various analyses have highlighted how CVD is becoming a leading threat for health in BC patients, especially in individuals older than 65 years or with pre-existing CV conditions (3–5). However, in common clinical practice, a focus on CV health in BC women is not routinely undertaken, and cardiologic involvement has been mostly dedicated to cardiotoxicity due to anticancer treatments.

Several BC therapies may indeed cause CV adverse effects, in the short and long-term, and in particular left ventricular dysfunction (LVD) due to anthracyclines and drugs targeting the human epidermal growth factor receptor 2 (HER2) (6, 7). The discipline of Cardio-Oncology was initially devoted to identification and management of cardiotoxicity (8). As traditional CV risk factors predispose to cardiotoxicity (9), and to adverse outcomes after oncologic treatment in cancer survivors (10), the need for CV prevention in oncology has emerged as another important issue (11). Nonetheless, integrated data about these diverse aspects of Cardio-Oncology practice are limited.

We herein present a monocentric experience of early cardiologic evaluation of BC patients in the setting of a structured Cardio-Oncology programme, not exclusively dealing with cardiotoxicity, but dedicated to the diversity of CV issues of BC women. Aim of the present study was to delineate a comprehensive view of CV risk profile assessment and management before anticancer treatment initiation, and occurrence of CV events after treatment in a contemporary cohort of BC patients.

## Methods

We retrospectively reviewed the records of all BC patients referred for a Cardio-Oncologic evaluation before starting anticancer treatment (i.e., baseline evaluation) at the IRCCS Ospedale Policlinico San Martino Cardio-Oncology Outpatient Clinic between 1st January, 2016 and 15th June, 2019. All patients were managed in accordance with the Declaration of Helsinki and signed an informed consent for the processing of personal data for scientific research purpose.

The Cardio-Oncologic evaluation consisted of collection of clinical history, physical examination, 12-lead ECG, and transthoracic echocardiography according to current guidelines (12). CV risk factors were assessed, and lifestyle changes and/or medications recommended if necessary.

The following data were extracted: (i) prevalence of CV risk factors, namely arterial hypertension, dyslipidaemia, smoking, diabetes mellitus and family history of coronary artery disease (CAD); (ii) control of CV risk factors, where inadequate control was defined as previously unknown arterial hypertension or known arterial hypertension with blood pressure (BP) values not at target (13), blood cholesterol levels not at target according to the CV risk profile (14), active smoking, or glycaemic values not at target (15); (iii) novel CVD diagnoses (i.e., any CVD unknown prior to baseline Cardio-Oncologic evaluation).

Since baseline evaluation, all patients were prospectively followed up with regular Cardio-Oncologic and/or oncologic evaluations. Follow up was censored at 15th June, 2020 or at time of death. No patients were lost at follow-up. The following outcomes were assessed: (i) LVD cardiotoxicity; (ii) CV events other than LVD occurring after initiation of anticancer therapy; (iii) all-cause mortality. LVD cardiotoxicity was defined as a drop in LV ejection fraction (EF) of >10% from baseline values and below 53%, according to the 2016 American Society of Echocardiography/European Association of Cardiovascular Imaging Expert Consensus (16). Other CV events were adjudicated based on medical reports.

### Statistical Analysis

Continuous variables, reported as mean ± SD or as median and minimum-maximum range for non-normal distributions, were compared by Student's *t*-test or Mann-Whitney test, as appropriate. Categorical variables, reported as percentages, were compared by chi-squared test. When feasible, Cox multivariable regression analysis (variable selection method: backward stepwise elimination) was performed including all candidate variables (*p* < 0.10 in univariate analysis). Incidence rates of events during follow-up were compared with Poisson regression analysis. A two-sided *p* < 0.05 was considered statistically significant. Data were analyzed with SPSS software version 25 (SPSS Inc., Chicago, Illinois).

## Results

Study population consisted of 508 women with BC. Their characteristics are shown in Table 1, and details about anticancer treatments in Supplementary Table 1. Mean age was 64 ± 13 years and 259 (51%) patients were >65 years old. The median number of Cardio-Oncologic evaluations per patient was 2 [1–15]. Two-hundred thirty-five (46%) patients received only one Cardio-Oncologic (i.e., baseline) evaluation.

**Table 1:** Characteristics of study population.

	***n* = 508 (%)**
Age (mean ± SD)	64 ± 13
Age >65 years	259 (51)
***Oncologic profile***	
Cancer setting	
Neoadjuvant	108 (21)
Adjuvant	295 (58)
Advanced	105 (21)
Previous exposure to anthracyclines	39 (8)
Anticancer treatment	
Anthracycline	287 (57)
Epirubicin	274 (54)
Cumulative dose (mean ± SD; mg/mq)	353 ± 68
Doxorubicin	3 (<1)
Liposomal	10 (2)
Anti*-*HER2	165 (33)
Trastuzumab	129 (25)
Trastuzumab and pertuzumab	36 (7)
Anthracyclines + anti-HER2	91 (18)
Cardio-Oncologic evaluations (median, [range])	2 [1–15]
***CV risk profile***	
Arterial hypertension	232 (46)
Inadequately controlled	94 (41)
Dyslipidaemia^*^	302 (59)
Inadequately controlled	270 (89)
Diabetes mellitus	47 (9)
Inadequately controlled	10 (21)
Tobacco smoking	185 (36)
Active	76 (41)
Former	109 (59)
Family history of CAD	63 (12)
≥2 CV risk factors	267 (53)
BMI > 30 kg/mq	69 (14)
Chronic kidney disease	16 (3)
Pre-existing CVD	
CAD	13 (3)
PAD	28 (6)
AF	23 (5)
HF	10 (2)
LVH	111 (22)
Moderate-to-severe VHD	20 (4)
Baseline LVEF	60 ± 3

* *In 59 patients, lipid blood values were not available and a diagnosis of dyslipidaemia was based solely on clinical history*.

*HER2, human epidermal growth factor receptor 2; CV, cardiovascular; CAD, coronary artery disease; PAD, peripheral artery disease; AF, atrial fibrillation; HF, heart failure; LVH, left ventricular hypertrophy; VHD, valvular heart disease; LVEF, left ventricular ejection fraction*.

### Baseline Cardio-Oncologic Evaluation

At the time of the baseline Cardio-Oncologic evaluation, 295 (58%) patients were in an adjuvant cancer setting (Table 1). Two-hundred eighty-seven (57%) were scheduled to receive anthracycline chemotherapy and 165 (33%) anti-HER2 targeted therapy, with trastuzumab alone (129, 25%) or trastuzumab and pertuzumab (36, 6%). Thirty-nine (8%) patients had a previous exposition to anthracyclines.

#### Baseline Cardiovascular Profile Assessment

Two-hundred thirty-two (46%) patients had arterial hypertension, 302 (59%) dyslipidaemia, 47 (9%) diabetes mellitus, 185 (36%) were smokers, and 63 (12%) had family history of CAD. Overall, 53% of patients had ≥2 CV risk factors. Pre-existing CVD was infrequent, with peripheral arterial disease (6%) and atrial fibrillation (AF, 5%) being the most common conditions. Inadequate control of CV risk factors was found in 94 hypertensive patients (41% of those with arterial hypertension), 270 dyslipidaemic patients (89%), and 10 diabetic patients (21%). Overall, 340 (67%) BC patients had at least one of these CV risk factors not adequately controlled. Moreover, 109 women were active smokers (59% of those with a history of smoking).

Mean LVEF was 60 ± 3%. One-hundred eleven (22%) patients had left ventricular hypertrophy; 99 (89%) of these patients were hypertensive, and 46 of them had BP values not at target.

#### Novel Cardiovascular Diagnoses

At baseline Cardio-Oncologic evaluation, 18 (4%) BC patients were found to have a previously unknown CVD: 6 moderate-to-severe valvular heart disease, 6 AF, 4 HF (2 with reduced EF and 2 with preserved EF), 1 CAD, and 1 a thoracic aortic aneurysm. Following these diagnoses, an appropriate treatment was started in each case; the oncologic therapeutic strategy did not change due to novel CVD diagnosis.

### Clinical Course After Initiation of Anticancer Treatment

**Figure 1:**
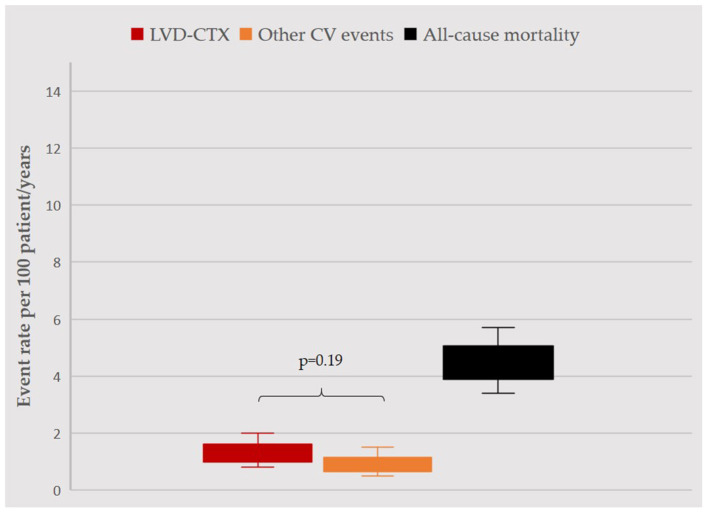
Incidence rate of events during follow-up. Incidence rates per 100 patient/years, with confidence intervals, for LVD (red), other CV events (orange) and mortality (black) are displayed. LVD and other CV events occurred with similar rates (*p* = 0.19). Rate of mortality was significantly higher as compared to other events (in both cases, *p* < 0.05), yet overall survival rate was >95%. For the purpose of this analysis, follow-up was censored at occurrence of any event (1207.2 patient/years). LVD, left ventricular dysfunction; CV, cardiovascular.

Over a mean follow-up of 2.5 ± 1 years, accounting for 1251.4 patient/years, 15 (3%) BC patients developed LVD, 10 (2%) suffered from other CV events and 55 (11%) died (Figure 1).

#### Left Ventricular Dysfunction

Mean time from baseline Cardio-Oncologic evaluation to LVD was 1 ± 0.9 years, and incidence rate was 1.2 per 100 patient/years (95% CI: 0.8–2.0).

Five patients also presented HF symptoms (33%). Mean LVEF at the time of LVD was 42 ± 6%. Of the 15 LVD events, 13 occurred during anti-HER2 therapy (7 patients treated with trastuzumab alone and 6 with trastuzumab and pertuzumab); 1 at the end of treatment with liposomal anthracycline and a cyclin-inhibitor; and 1 as a late asymptomatic LVD 3.5 years after treatment with anthracycline, cyclophosphamide, 5-fluoro-uracil, and taxanes.

**Figure 2:**
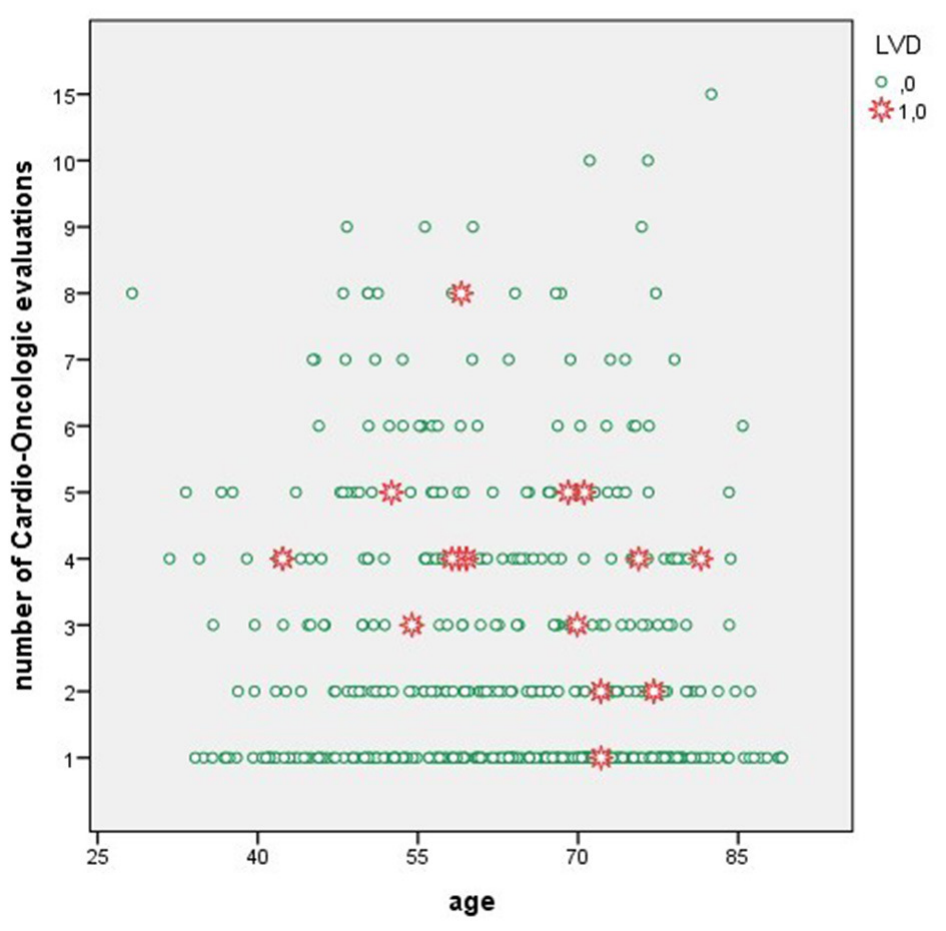
Distribution of LVD events according to number of Cardio-Oncologic evaluations.

Characteristics of BC patients with and without LVD are reported in Table 2. Those who developed LVD were more commonly treated with anti-HER2 therapies (87 vs. 31%, *p* < 0.001) and with anthracyclines in combination with anti-HER2 (53 vs. 17%, *p* = 0.002). They also received more Cardio-Oncologic evaluations (4 [1–8] vs. 2 [1–15], *p* = 0.001, Figure 2) and had more frequently a family history of CAD (40 vs. 12%, *p* = 0.001).

**Table 2:** Characteristic of BC patients with and without LVD.

	**Overall population (*****n*** **=** **508)**			**Anti-HER2 population (*****n*** **=** **165)**		
**Variable**	**With LVD**	**Without LVD**	***p***	**With LVD**	**Without LVD**	***p***
	***n =* 15 (%)**	***n =* 493 (%)**		***n =* 13 (%)**	***n =* 152 (%)**	
Age (mean ± SD)	65 ± 11	64 ± 13	0.67	66 ± 11	61 ± 13	0.19
Age >65 years	8 (53)	251 (51)	1	8 (62)	64 (42)	0.25
Cancer setting			0.14			0.20
Neoadjuvant	5 (33)	103 (21)		5 (39)	45 (30)	
Adjuvant	5 (33)	290 (59)		4 (31)	83 (55)	
Advanced	5 (33)	100 (20)		4 (31)	24 (16)	
Previous exposure to anthracyclines	1 (7)	38 (8)	1	1 (8)	6 (4)	0.44
**Anticancer treatments**						
Anthracyclines	10 (67)	277 (56)	0.60	8 (62)	83 (55)	0.77
Cumulative dose (mean ± SD; mg/mq)	315 ± 42	354 ± 68	0.11	315 ± 42	343 ± 45	0.09
Anti-HER2	13 (87)	152 (31)	**<0.001**	-	-	-
Trastuzumab	7 (47)	122 (25)	0.07	-	-	-
Trastuzumab and pertuzumab	6 (40)	30 (6)	**<0.001**	6 (46)	30 (20)	**0.04**
Anthracyclines+anti-HER2	8 (53)	83 (17)	**0.002**	-	-	**-**
Cardio-Oncologic evaluations (median, [range])	4 [1–8]	2 [1–15]	**0.001**	4 [2–8]	4 [1–15]	0.43
Arterial hypertension	9 (60)	223 (45)	0.30	8 (62)	63 (41)	0.24
Dyslipidaemia	9 (60)	293 (59)	1	7 (54)	82 (54)	1
Diabetes mellitus	3 (20)	44 (9)	0.15	2 (15)	12 (8)	0.30
Tobacco smoking	7 (47)	178 (36)	0.42	6 (47)	54 (36)	0.55
Active	5 (33)	71 (14)	0.06	4 (31)	20 (13)	0.10
Family history of CAD	6 (40)	57 (12)	**0.001**	6 (46)	17 (11)	**0.003**
≥2 CV risk factors	10 (67)	257 (52)	0.30	9 (69)	71 (47)	0.15
BMI > 30 kg/mq	1 (7)	68 (14)	0.71	1 (8)	18 (12)	1
Chronic kidney disease	0 (0)	16 (3)	1	0 (0)	4 (3)	1
**Known CV conditions**						
CAD	2 (13)	11 (2)	0.06	2 (15)	4 (3)	0.07
PAD	1 (7)	27 (6)	0.58	1 (8)	7 (5)	0.49
AF	1 (7)	22 (5)	0.51	1 (8)	6 (4)	0.44
HF	1 (7)	9 (2)	0.26	1 (8)	2 (1)	0.22
LVH	3 (20)	108 (22)	1	3 (23)	22 (15)	0.42
Moderate-to-severe VHD	1 (7)	19 (4)	0.46	1 (8)	6 (4)	0.44
Baseline LVEF	57 ± 8	60 ± 2	0.14	56 ± 8	60 ± 2	0.11

*BC, breast cancer; LVD, left ventricular dysfunction; HER2, human epidermal growth factor receptor 2; CAD, coronary artery disease; CV, cardiovascular; PAD, peripheral artery disease; AF, atrial fibrillation; HF, heart failure; LVH, left ventricular hypertrophy; VHD, valvular heart disease; LVEF, left ventricular ejection fraction. The bold values highlight significant p values*.

Since the vast majority of BC patients had LVD during anti-HER2 therapy, characteristics of patients with and without LVD in this specific subgroup were compared (Table 2). Mean time from baseline Cardio-Oncologic evaluation to LVD among anti-HER2 recipients was 0.8 ± 0.4 years. Incidence rate was 3.2 per 100 patient/years (95% CI: 1.9–5.4). Of the 13 anti-HER2 LVD (4 with overt HF), 8 patients were also treated with anthracyclines, and 8 had recovery of LVEF (i.e., returning to baseline values).

BC patients with LVD due to anti-HER2 were more commonly treated with pertuzumab (46 vs. 20%, *p* = 0.04), but there was no association with anthracyclines therapy (62 vs. 55%, *p* = 0.77). CV profile was similar between the two groups except for family history of CAD (46 vs. 11%, *p* = 0.003).

#### Other Cardiovascular Events

Mean time from baseline Cardio-Oncologic evaluation to CV events other than LVD was 1.1 ± 0.8 years, and incidence rate was 0.8 per 100 patient/years (95% CI: 0.5–1.5).

Details of CV events are reported in Supplementary Table 2. Three BC patients had an episode of pulmonary embolism; 2 (treated with anti-vascular endothelial growth factor agents) developed uncontrolled arterial hypertension; 2 were found to have AF; 1 had cardiac tamponade; 1 an episode of takotsubo syndrome and 1 a fatal ischaemic stroke (late after anticancer treatment completion). Anticancer therapy was permanently interrupted only in one pulmonary embolism case.

Characteristics of patients with and without CV events other than LVD are shown in Table 3. Those with events were slightly older (*p* = 0.07), and more commonly in an advanced cancer setting (50%), while those without were in an adjuvant setting (59%, *p* = 0.03). There were no differences in terms of anticancer therapies, but those with CV events more frequently had a previous exposition to anthracyclines (30 vs. 7%, *p* = 0.03). Patients with events had more commonly arterial hypertension (80 vs. 45%, *p* = 0.05) and LVH (50 vs. 21%, *p* = 0.05).

**Table 3:** Characteristic of BC patients with and without CV events other than LVD.

**Variable**	**With events *n =* 10 (%)**	**Without event *n = 498 (%)***	***p***
Age (mean ± SD)	71 ± 12	63 ± 13	0.07
Age >65 years	7 (70)	252 (51)	0.34
Cancer setting			**0.03**
Neoadjuvant	3 (30)	105 (21)	
Adjuvant	2 (20)	293 (59)	
Advanced	5 (50)	100 (20)	
Previous exposure to anthracyclines	3 (30)	36 (7)	**0.03**
**Anticancer treatments**			
Anthracyclines	4 (40)	283 (57)	0.34
Cumulative dose (mean ± SD; mg/mq; not liposomal)	409 ± 180	352 ± 65	0.57
Anti-HER2	2 (20)	163 (33)	0.51
Only trastuzumab	1 (10)	128 (26)	0.46
Trastuzumab and pertuzumab	1 (10)	35 (7)	0.52
Anthracyclines+anti-HER2	2 (20)	89 (18)	0.70
Number of Cardio-Oncologic evaluations (median, [range])	3 [1–9]	2 [1–15]	0.20
Arterial hypertension	8 (80)	224 (45)	**0.05**
Dyslipidaemia	7 (70)	295 (59)	0.75
Diabetes mellitus	1 (10)	46 (9)	1
Tobacco smoking	3 (30)	182 (37)	0.75
Active	1 (10)	75 (15)	1
Family history of CAD	1 (10)	62 (12)	1
≥2 CV risk factors	7 (70)	260 (52)	0.35
BMI > 30 kg/mq	1 (10)	68 (14)	1
Chronic kidney disease	0 (0)	16 (3)	1
**Known CV conditions**			
CAD	0 (0)	13 (3)	1
PAD	0 (0)	28 (6)	1
AF	1 (10)	22 (4)	0.37
HF	0 (0)	12 (2)	1
LVH	5 (50)	106 (21)	**0.05**
Moderate-to-severe VHD	0 (0)	20 (4)	1
Mean LVEF	60 ± 3	60 ± 0	0.73
LVD-CTX	0 (0)	15 (3)	1

*BC, breast cancer; HER2, human epidermal growth factor receptor 2; CV, cardiovascular; CAD, coronary artery disease; PAD, peripheral artery disease; AF, atrial fibrillation; HF, heart failure; LVH, left ventricular hypertrophy; VHD, valvular heart disease; LVEF, left ventricular ejection fraction; LVD, left ventricular dysfunction. The bold values highlight significant p values*.

#### All-Cause Mortality

Mean time from baseline Cardio-Oncologic evaluation to death was 1.5 ± 1.1 years, and incidence rate was 4.4 per 100 patient/years (95% CI: 3.4–5.7); with overall survival rate ranging 95–97% per year.

As shown in Table 4, BC patients who died were older (68 ± 11 vs. 63 ± 13 years, *p* = 0.01), with advanced cancer (64 vs 16%, *p* < 0.001) and more frequently with a previous exposition to anthracyclines (24 vs. 7%, *p* < 0.001). AF was more common among those who died (13 vs. 4%, *p* = 0.01).

**Table 4:** Characteristics of BC patients who died at follow-up and who survived.

**Variable**	**Died** ***n =* 55 (%)**	**Survived** ***n =* 453 (%)**	***p***
Age (mean ± SD)	68 ± 11	63 ± 13	**0.01**
Age >65 years	36 (66)	223 (49)	**0.03**
Cancer setting			**<0.001**
Neoadjuvant	9 (16)	99 (22)	
Adjuvant	11 (20)	284 (63)	
Advanced	35 (64)	70 (16)	
Previous exposure to anthracyclines	13 (24)	26 (6)	**<0.001**
**Anticancer treatments**			
Anthracyclines	16 (29)	271 (60)	**<0.001**
Cumulative dose (mean ± SD; mg/mq; not liposomal)	344 ± 89	353 ± 67	0.59
Anti-HER2	10 (18)	155 (34)	**0.02**
Only trastuzumab	4 (7)	125 (28)	**<0.001**
Trastuzumab and pertuzumab	6 (11)	30 (7)	0.26
Anthracyclines+anti-HER2	3 (6)	88 (19)	**0.01**
Number of Cardio-Oncology evaluations (median, [range])	2 [1–15]	2 [1–10]	0.74
Arterial hypertension	32 (58)	200 (44)	0.06
Dyslipidaemia	33 (60)	269 (59)	1
Diabetes mellitus	9 (16)	38 (8)	0.08
Tobacco smoking	20 (36)	165 (36)	1
Active	9 (16)	67 (15)	0.69
Family history of CAD	5 (9)	58 (13)	0.52
≥2 CV risk factors	32 (59)	235 (52)	0.40
BMI > 30 kg/mq	7 (13)	62 (14)	1
Chronic kidney disease	2 (4)	14 (3)	0.69
**Known CV conditions**			
CAD	2 (4)	11 (2)	0.64
PAD	5 (9)	23 (5)	0.21
AF	7 (13)	16 (4)	**0.01**
HF	2 (4)	8 (2)	0.30
LVH	16 (29)	95 (21)	0.17
Moderate-to-severe VHD	3 (6)	17 (4)	0.47
Mean LVEF	59 ± 3	60 ± 3	0.24
LVD	0 (0)	15 (3)	0.39
Other CV events	4 (7)	6 (1)	**0.02**

*BC, breast cancer; HER2, human epidermal growth factor receptor 2; CV, cardiovascular; CAD, coronary artery disease; PAD, peripheral artery disease; AF, atrial fibrillation; HF, heart failure; LVH, left ventricular hypertrophy; VHD, valvular heart disease; LVEF, left ventricular ejection fraction; LVD, left ventricular dysfunction. The bold values highlight significant p values*.

No LVD events occurred among BC patients who died, whereas CV events other than LVD were significantly more common (7 vs. 1%, *p* = 0.02). At multivariate Cox regression analysis (Supplementary Table 3), only an advanced cancer setting (HR 7.80 [4.47–13.62], *p* < 0.001) and AF (HR 4.12 [1.85–9.20], *p* = 0.001) were significantly associated with the risk of death.

## Discussion

CV health in BC women is a matter of concern for several reasons: awareness toward CV prevention in females is suboptimal; management of CV risk profile may be overlooked in oncologic patients; anticancer therapies may cause or predispose to CV events, affecting cancer survivorship (2, 17). However, in BC patients, cardiologic attention and involvement is mostly limited to detection and management of LVD due to anthracyclines and anti-HER2 therapies, and an integrated approach caring for bidirectional CV and oncologic needs is often lacking. At our Institution, a baseline cardiologic evaluation in the setting of a structured Cardio-Oncology programme helped assessing this gap in clinical practice and appreciating the diversity of CV issues which may affect BC women, beyond the sole cardiotoxicity.

### Usefulness of a Baseline Cardio-Oncologic Evaluation for Cardiovascular Prevention

In this BC cohort, prevalence of CV risk factors was significant, and higher as compared to the European general population (18, 19). Most importantly, CV risk factors control was suboptimal, in particular in the case of dyslipidaemia and arterial hypertension. Overall, the baseline Cardio-Oncologic evaluation allowed to implement CV prevention, recognize unknown CV conditions (even severe valvular heart disease or CAD) and optimize CV profile in the vast majority of BC patients. In a contemporary American BC population receiving trastuzumab therapy, cardiologic involvement during oncologic treatment was performed in <30% of cases (20). BC patients undergoing cardiologic evaluations, however, showed improvements in CV risk factor control.

The usefulness of a baseline cardiologic consultation, in terms of assessment of LVEF, in BC patients scheduled to receive anthracycline treatment, has been questioned given that detection of reduced LVEF, and therefore indications for changing chemotherapy strategy, is generally low (21). However, in contemporary care, Cardio-Oncology should not be intended as a simple act of cardiologic clearance to anticancer treatments, rather it should encompass a thorough assessment of CV risk profile (6). Moreover, for many oncologic patients, a baseline Cardio-Oncologic evaluation may represent the first (and even the only, as was the case for several of the BC women in our cohort) occasion to undergo a cardiologic consultation. With non-adherence to CV medications being detrimental for long-term outcome of BC patients, the baseline Cardio-Oncologic evaluation may also be the chance for CV health education and motivational support (20, 22). It is undeniable that such an “holistic” cardiologic approach requires resources and may be perceived as time consuming. Nevertheless, it is reasonable to assume that the implementation of CV prevention in the oncologic setting by the means of Cardio-Oncology would be of great potential, given the high prevalence of CV comorbidity and risk factors, especially in women undertreated with little awareness of their CV risk (1, 2, 4, 11). Moreover, a well-delivered baseline cardiologic assessment may avoid unplanned CV evaluations during the anticancer treatment period.

### Insights into Left Ventricular Dysfunction Due to Anticancer Treatment

Few LVD events occurred in our cohort. Contrary to previous reports (23, 24), classic modifiable CV risk factors, and in particular arterial hypertension, were not associated with the occurrence of LVD. It might be speculated that such finding was the straight consequence of CV risk profile optimization secondary to the baseline Cardio-Oncologic evaluation. Since the risk of cardiotoxicity is mainly related to the individual baseline CV risk profile and the intrinsic toxicity of a given drug (9), “blunting” the CV profile may result in reducing cardiotoxicity risk. In a cohort of oncologic patients treated with anti-vascular endothelial growth factor agents, we have shown that an approach based on a structured Cardio-Oncology programme with baseline evaluation and tailored recommendations for BP management resulted in a lack of association between both controlled and uncontrolled arterial hypertension at baseline and the risk of CV events (25). Similarly, in this BC population, modifiable CV risk factors were not associated with LVD occurrence and its risk was mostly driven by the inherent toxicity of anticancer treatments, such as combined anti-HER2 therapy. Taken together, these findings highlight the importance of baseline CV evaluation of BC women, and that of adequate monitoring of patients scheduled to receive at-risk treatments, even in the long-term (6, 7). However, it should be noted that BC patients with LVD in the overall study population underwent a greater number of Cardio-Oncologic evaluation as compared to those without LVD (Figure 2), while this result was not found in the anti-HER2 subgroup, likely due to the regular echocardiographic monitoring these patients undergo. Moreover, only a third of patients with LVD had overt HF, and more than a half had complete recovery of LVEF. Analyses from the *Cardiotoxicity of Cancer Therapy study* showed that BC patients treated with anthracyclines and/or anti-HER2 therapies frequently display subclinical, modest, but persistent indexes of LVD, with only partial recovery over time (26, 27). However, association of LVD with overt HF is unclear, and studies to understand the long-term significance of modest-but-stable systolic dysfunction are needed (27). Moreover, a recent case-control study of BC patients treated with trastuzumab showed no association between adherence to echocardiographic monitoring and risk of HF (23). Thus, it may be worth to re-evaluate clinical practice in the light of novel findings, in order to understand what would be the best strategy to follow-up BC patients, especially if treated with anti-HER2 therapy, prior, during and after anticancer treatment. In other words, the risk of cardiotoxicity related to this anticancer treatment does not appear to be reduced by a careful echocardiographic monitoring. Strategies differing from the 3/6 months echocardiographic LVEF screening, derived from seminal trials (28), may be worth of investigation in future studies.

Finally, it is of note that the only CV risk factor significantly associated with LVD was family history of CAD. It has been found that sarcomeric gene variants contribute to the risk of developing cancer-therapy induced cardiomyopathy (mostly due to anthracyclines) (29). Our data may indicate that at some extent a genetic background predisposition might also contribute to the development of early LVD due to anti-HER2 therapy, warranting further investigations.

### The Burden of Cardiovascular Events Other than Left Ventricular Dysfunction

Beyond LVD, there is a broad spectrum of CV issues that may affect BC women. These CV events are not of secondary importance in the clinical history of BC women, as they may be severe and happen with a similar rate to that of LVD (Figure 1). Some of these CV events were clearly related to anticancer therapy (as elevation of BP with anti-vascular endothelial growth factor agents), while other may have been not, and just have resulted from an unfavorable CV profile. Indeed, characteristics of BC patients experiencing these CV events were different from those of patients with LVD. BC women with CV events other than LVD had more commonly advanced cancer (and as such had more frequently a previous exposition to anthracyclines) and a worse CV risk profile, characterized by arterial hypertension with organ damage. The occurrence of these CV events may be the result of the interaction of cardiologic and oncologic comorbidities, which may intersect and favor occurrence of adverse clinical outcomes (2, 30). Consistently, BC patients who did not survive, as compared to survivors, more frequently experienced a CV event other than LVD during their clinical course. In other words, the burden of CV risk factors and CVD extend beyond the risk of LVD, and has a significant impact on the clinical course of oncologic patients (31). As a plausible consequence, in our cohort, AF was the only predictor of mortality – together with advanced cancer, as expected –, reflecting the fact that AF represents a proxy of frailty (32).

All these aspects underline the importance of CV prevention in oncologic patients, and specifically in BC women. Adequate CV risk profile optimization and management exerts beneficial effects not only in the short-term to minimize cardiotoxicity, but also in the long-term clinical course, both from the cardiologic and the oncologic standpoint (2, 11). This consideration acquires even more importance when one considers that mortality was overall low, and therefore the vast majority of our BC cohort would experience extended life expectancy. In such a perspective, CV prevention becomes of paramount importance.

## Limitations

This is a monocentric retrospective study in which BC women with a baseline Cardio-Oncologic evaluation were evaluated. Such approach is not mandatory in our Institution and thus, though being a minority of cases, some patients might have not undergone a baseline Cardio-Oncologic evaluation. This may partially have represented a selection bias. Moreover, being a retrospective real-world analysis, in our analysis a comparator group is not present (i.e., BC women not undergoing Cardio-Oncologic evaluation). The low number of LVD and other CV events in our cohort may have influenced comparisons between groups with and without events (Tables 2, 3). These results are only of association, should be considered hypothesis-generating and interpreted with caution. Given all these aspects, survival analyses were not performed for these events. Data regarding hormonal therapy and radiotherapy were incomplete and therefore were not included in the analysis.

We recognize these shortcomings of our work. However, to our knowledge, few studies have previously comprehensively assessed CV health of BC women in large cohorts, as we did.

## Conclusion

BC women show a suboptimal CV risk profile before initiation of anticancer treatments and during their clinical course are at risk of experiencing CV events not limited to LVD. In this cohort, a baseline Cardio-Oncologic evaluation was instrumental to deal with all these aspects, by implementing CV education and prevention strategies, and by optimizing CV therapies when needed. Bearing in mind CV health of BC women since the beginning of oncologic treatment is likely to exert beneficial effects in the short and long-term.

## Data Availability Statement

The raw data supporting the conclusions of this article will be made available by the authors, without undue reservation.

## Author Contributions

GT and PS conceived the idea. GT, PA, MS, and PS developed the project. GT, GB, MS, and GG collected data. GT performed statistical analysis. GT wrote the initial draft with the help of PA, PS, and IP. All authors critically revised the manuscript and agreed with the final version.

## Conflict of Interest

The authors declare that the research was conducted in the absence of any commercial or financial relationships that could be construed as a potential conflict of interest.

## Publisher's Note

All claims expressed in this article are solely those of the authors and do not necessarily represent those of their affiliated organizations, or those of the publisher, the editors and the reviewers. Any product that may be evaluated in this article, or claim that may be made by its manufacturer, is not guaranteed or endorsed by the publisher.
