# Supplementary material for: Diversity of Cardiologic Issues in a Contemporary Cohort of Women With Breast Cancer
Source: Front Cardiovasc Med. 2021 Oct 1;8:654728. doi: 10.3389/fcvm.2021.654728 (PMC8517118; doi:10.3389/fcvm.2021.654728)
Supplement: Supplementary file 1 [file Data_Sheet_1.pdf]

Supplemental Material

Tini et al., *Diversity of Cardiologic Issues in a Contemporary Cohort of Women with Breast Cancer*

Supplementary Table 1s. Details of anticancer treatments

	n = 508 (%)
Anthracyclines	287 (57)
In combination with anti-HER2	91 (18)
In combination with taxanes	257 (51)
In combination with pyrimidines	29 (6)
In combination with ciclophosphamide	275 (54)
Anti-HER2	165 (33)
Only trastuzumab	129 (25)
Trastuzumab and pertuzumab	36 (7)
In combination with taxanes	148 (29)
Taxanes & Ciclophosphamide	
In combination with other therapies	291 (57)
Alone	32 (6)
Pyrimidines	79 (16)
Cycline-inhibitors	46 (9)
Anti-VEGF	16 (3)

VEGF: vascular endothelial growth factor; HER2: human epidermal growth factor receptor 2

Supplementary Table 2s. Details of CV events other than LVD-CTX

Event	n (10)	Anticancer treatment
Pulmonary Embolism	3	Epirubicin (1)<br>Epirubicin, cyclophosphamide and 5-fluoro-uracil (1)<br>Capecitabine (1)
Uncontrolled arterial hypertension	2	Anti-VEGF
Atrial fibrillation	2	Cyclin-inhibitors (1)<br>Taxanes (1)
Cardiac tamponade	1	Anti-HER2 (trastuzumab and pertuzumab)
Takotsubo syndrome	1	Cyclin-inhibitors
Ischaemic stroke	1	Epirubicin, cyclophosphamide, taxanes and trastuzumab (1 year after completion)

CV: cardiovascular; LVD-CTX: left ventricular dysfunction cardiotoxicity; VEGF: vascular endothelial growth factor; HER2: human epidermal growth factor receptor 2

Supplementary Table 3s. Predictors of all-cause mortality

	Univariate		Multivariate*	
Variable	*HR (95%CI)*	*p*	*HR (95%CI)*	*p*
Age	1.04 (1.01-1.06)	0.004	-	-
**Advanced setting**	**7.76 (4.48-13.45)**	**<0.001**	**7.80 (4.47-13.62)**	**<0.001**
Previous exposition to anthracyclines	4.12 (2.20-7.70)	<0.001	-	-
Anthracycline chemotherapy	0.28 (0.16-0.50)	<0.001	-	-
Trastuzumab therapy	0.21 (0.08-0.59)	0.003	-	-
Arterial hypertension	1.75 (1.03-3.01)	0.04	-	-
Diabetes mellitus	2.12 (1.04-4.34)	0.04	-	-
**AF**	**3.42 (1.55-7.56)**	**0.002**	**4.12 (1.85-9.20)**	**0.001**
**Other CV events**	**3.84 (1.39-10.63)**	**0.01**	1.77 (0.63-4.95)	0.28

*variable selection method: backward stepwise elimination

AF: atrial fibrillation; CV: cardiovascular
